# Stability of Bivariate GWAS Biomarker Detection

**DOI:** 10.1371/journal.pone.0093319

**Published:** 2014-04-30

**Authors:** Justin Bedő, David Rawlinson, Benjamin Goudey, Cheng Soon Ong

**Affiliations:** 1 NICTA Victoria Research Laboratory, University of Melbourne, Victoria, Australia; 2 Department of Computing and Information Systems, University of Melbourne, Victoria, Australia; 3 Department of Electrical & Electronic Engineering, University of Melbourne, Victoria, Australia; Shenzhen Institutes of Advanced Technology, China

## Abstract

Given the difficulty and effort required to confirm candidate causal SNPs detected in genome-wide association studies (GWAS), there is no practical way to definitively filter false positives. Recent advances in algorithmics and statistics have enabled repeated exhaustive search for bivariate features in a practical amount of time using standard computational resources, allowing us to use cross-validation to evaluate the stability. We performed 10 trials of 2-fold cross-validation of exhaustive bivariate analysis on seven Wellcome–Trust Case–Control Consortium GWAS datasets, comparing the traditional 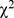 test for association, the high-performance GBOOST method and the recently proposed GSS statistic (Available at http://bioinformatics.research.nicta.com.au/software/gwis/). We use Spearman's correlation to measure the similarity between the folds of cross validation. To compare incomplete lists of ranks we propose an extension to Spearman's correlation. The extension allows us to consider a natural threshold for feature selection where the correlation is zero.

This is the first reported cross-validation study of exhaustive bivariate GWAS feature selection. We found that stability between ranked lists from different cross-validation folds was higher for GSS in the majority of diseases. A thorough analysis of the correlation between SNP-frequency and univariate 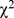 score demonstrated that the 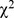 test for association is highly confounded by main effects: SNPs with high univariate significance replicably dominate the ranked results. We show that removal of the univariately significant SNPs improves 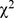 replicability but risks filtering pairs involving SNPs with univariate effects. We empirically confirm that the stability of GSS and GBOOST were not affected by removal of univariately significant SNPs.

These results suggest that the GSS and GBOOST tests are successfully targeting bivariate association with phenotype and that GSS is able to reliably detect a larger set of SNP-pairs than GBOOST in the majority of the data we analysed. However, the 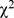 test for association was confounded by main effects.

## Introduction

Genome-Wide Association Studies (GWAS) measure hundreds of thousands of SNPs from thousands of individuals with the aim of detecting statistical association between individuals' phenotype and genotype. SNPs are known to be useful markers for disease and are typically measured using microarray-based approaches [1]. The most common GWAS designs are Case-Control studies of human disease, where the phenotype of each individuals is a binary label indicating the presence or absence of disease; these individuals are called cases or controls respectively.

Existing research has identified a number of SNPs that are believed to confer an increased or reduced risk of disease [2]. However, despite application of numerous methods to GWAS, for most diseases there remains a gap between the level of association observed from the SNPs and the total level of genetic heritability known to exist; this is the problem of “missing heritability” [3]. One hypothesis is that the missing heritability of disease phenotypes could be further explained by combinatorial analysis of interactions between SNPs [4]. However, there are few studies that have demonstrated interactions between SNPs that replicate across multiple datasets, let alone explaining some portion of the missing heritability.

Historically, computational complexity has made combinatorial SNP analysis infeasible. As a typical GWAS study contains over 500,000 SNPs, exhaustive searching for interactions between pairs of SNPs requires that more than 125 billion pairs are considered. Since the number of interactions considered grows exponentially with the size of the interaction, exhaustive interaction analysis is likely to remain infeasible for more complex interactions of 4th order or more. However, recent methods have been developed that are able to perform exhaustive two-way analysis in a reasonable amount of time [5], [6], [7], [8]. Difficulties with this type of analysis remain, with recently published data showing that attempts to use conventional tests of association to select bivariate effects may be confounded by univariate effects [5], indicating that statistical issues are also preventing effective use of GWAS for the understanding of disease biology.

From a machine learning perspective, Case–Control GWAS studies can be modelled as a binary classification or regression problem. The task of identifying meaningful SNPs is essentially a feature selection task [9], and the search for higher order interaction amounts to simultaneously finding multiple explanatory variables. We compare three approaches for identifying bivariate features: 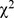 test of association corresponding to a traditional feature selection approach, and two recently published methods GSS [5] and GBOOST [10] corresponding to the binary classification and regression setting respectively.

The approach we take in this paper is variable ranking, and we focus on bivariate features. This is a natural extension to the univariate analysis (studying individual SNPs) that has already been performed [11], [12]. Motivated by recent work on gene expression data [13], [14] and univariate GWAS analysis [15], [16] that identifies *stable* features as good features, we perform cross validation to look for bivariate features that are stable when subsets of individuals are removed from the dataset.

### 1.1 Stability and replicability

It is hypothesised that networks of interacting alleles are responsible for some part of individuals' susceptibility to disease due to effects on a variety of cellular mechanisms [17]. However, discovery of such networks is in its infancy. Consequently, we do not possess a set of known SNP interactions that can be used to validate multivariate SNP detection techniques.

A common approach to testing interaction detection methods is to use simulated data, whereby specific causal relationships are inserted into randomly generated datasets, and methods' ability to recover the signal are measured. However, much is unknown about the structure of GWAS data and making it difficult to know whether the way in which data has been modelled is representative of interactions in real data. For example, it is unknown whether phenotypic consequences occur incrementally or suddenly given varying subsets of causal variants and the levels of risk these variants incur [4], [18]. While some attempts have been made to model such complexities, the validity of simulated data is currently unclear.

Given these concerns, we chose to measure the replicability of SNP-pair rankings on real GWAS data [19]. While only some consistently selected SNP-pairs might have a biological relationship with phenotype, any good pair-selection algorithm should reliably detect SNP-pairs that predict phenotype. Therefore, we critically investigated the replication results we obtained in an attempt to characterise the qualities of replicating pairs.

Interacting SNP pairs are commonly referred to as epistatic, though the precise definition of this term can vary greatly [20]. In the work conducted here, we do not search specifically for epistatic SNP pairs, given the complexity of this terminology. Instead we search for bivariate association with phenotype: combinations of SNPs that result in a stronger level of association than if either SNP were considered independently. This overlaps with some of the numerous definitions of epistasis but is potentially inconsistent with others [20]. Regardless of any underlying biological cause, pairs of SNPs that result in improved association with phenotype compared to use of these SNPs alone may improve estimates of heritability [4] and could be useful markers for clinical prediction of disease.

In this paper, we investigate 2 fold cross-validation where in each random split, individuals are separated into two equal sized subsets each containing all SNPs. For each pair of subsets, we apply a bivariate GWAS approach and determine whether the rankings of SNP pairs by the given statistic are consistent. This is motivated by a common approach in biology of replicating studies in two separate cohorts. Two fold cross validation simulates two equal sized cohorts of individuals which have had the same SNPs genotyped. An alternative resampling scheme is the bootstrap [21] method, however we chose to use cross-validation as it matches more closely the traditional multi-cohort design. While such an approach does not simulate the effects of measurement noise or population stratification between datasets, SNPs that are ranked differently across folds may be due to some bias in either the underlying statistical test or within the datasets under examination.

### 1.2 Genome Wide Interaction Search

Genome-Wide Interaction Search (GWIS) is a fast software program for detecting statistical association between pairs of SNPs and a given phenotype in GWAS data [5]. GWIS exploits statistics such as GSS that are specifically designed to search for an *improvement* in bivariate (SNP-pair) association with phenotype over the univariate association (individual SNPs). Unlike common regression-based approaches, these tests make no assumptions about the way in which disease risk is distributed amongst the genotypes for a given pair.

The GSS method used in this paper uses classification models to predict phenotype from genotype. For a given SNP pair, determining the GSS requires solving a non-trivial min-max optimisation problem (see the Methods section). Solving this optimisation problem efficiently enough to allow exhaustive analysis is difficult. Indeed, earlier benchmarks had suggested that bivariate exhaustive GSS on a typical dataset could take years to execute, hence Goudey et al. [5] were only able to apply GSS to top-ranked pairs from a pre-filtering heuristic. However, parallel implementation on NVIDIA's CUDA [22] General-Purpose Graphics Processing Unit (GPGPU) architecture reduced this runtime to approximately 6 hours, enabling us to perform cross-validation on GWAS data using the GSS method.

In this paper we further evaluate two existing methods for bivariate feature selection. The most widespread alternative to classification is regression, in which genotypes are used as explanatory variables and phenotype as a dependent variable. A popular example of such a method is BOOST [10] and its GPU implementation GBOOST [23].

Like GWIS, BOOST measures the improvement over the effects of the marginals but the approach is fundamentally different: BOOST is grounded in traditional statistics and uses a likelihood ratio test to reject the hypothesis that the interaction term does not improve the model (i.e., that the SNP-pair does not improve linear combinations of the marginals). We include GBOOST in our study as a representative from the family of regression methods (see the Methods section).

Pearson's 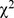 test for association is used as a representative of simpler statistics that do not explicitly detect interactions. Instead, the 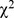 test looks for associations with phenotype that may include interactions between SNPs.

### 1.3 Measuring overlap in top-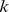 ranked lists

There are a number of difficulties measuring overlap between ranked lists, particularly for Case-Control GWAS that have categorical genotype and phenotype. With the number of individuals only in the thousands, the potential for tied scores is significant and ordering of equal scores is at best random. The top 500,000 ranks are likely to contain thousands of SNPs with equal score, and all tied scores will have an ordering unrelated to their significance. A good rank comparison algorithm should account for tied scores. The two common approaches for comparing ranked data are known as the Spearman's 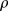 (Spearman Rank Correlation or Spearman's Rho) and Kendall's Tau (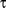).

Most importantly, when considering the ranks of features computed by variable ranking approaches, only meaningful features would be expected to have consistent ranks between different subsets of the data [24]. Features which do not contribute to explaining the phenotype would have an arbitrary rank, and hence would not be stable. Therefore it is desirable that comparisons between ranked lists of discovered features consider order stability in addition to the common elements.

Furthermore, ranked SNP-pairs are indefinite and incomplete lists [25]. The number of SNP-pairs with a causal relationship to phenotype is unknown, so it is difficult to determine the number of ranks in which statistical tests should be compared. For example, if only 30 SNP-pairs have any effect on phenotype, it is inappropriate to use the overlap in the top 100 ranked scores as a measure of test performance. As exhaustive bivariate analysis of a typical GWAS will examine billions of SNP pairs, it is impractical to record the rank of every pair. Instead, only a subset of top-ranked pairs will be recorded. Hence, the resulting list is incomplete as only a subset of all pairs are included in the ranked results.

In this paper we propose an extension of Spearman's 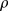 which compares two (incomplete) lists of top ranked objects. This takes the issues listed above into account and is described in the Methods section. In addition, we investigate the rank at which crosses zero (indicating no correlation) which we call Zero Index Crossing (ZIC), as a way to identify SNP pairs that are stable with respect to cross validation.

### 1.4 Related tasks and settings

In this paper we propose an extension of Spearman's 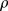 which compares two (incomplete) lists of top ranked objects. This takes the issues listed above into account and is described in the Methods section. In addition, we investigate the rank at which 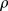 crosses zero (indicating no correlation) which we call Zero Index Crossing (ZIC), as a way to identify SNP pairs that are stable with respect to cross validation.

Our choice of Spearman's 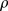 as the metric to measure the stability between two lists is motivated by the belief that the ordering is also an important aspect to stability in addition to the retrieved items. Spearman's 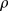 is particularly attractive as it has a strong theoretical basis and has been well studied. Other approaches in the literature such as have been motivated by specific applications, for example gene expression [26], and are not applicable in our setting.

Furthermore, there is a closely related problem of rank aggregation [27] where a set of stable objects are sought. Our approach does not directly result in informing which objects are stable, but does suggest what the size of such a set might be.

### 1.5 Contributions

The contributions of this paper are: examining stability of SNP-pairs discovered by exhaustive bivariate GWAS conducted in cross-validation, including the recently published GSS statistic compared to two reference methods, 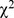 and GBOOST; novel insights into the stability performance of bivariate analysis using the 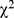 statistic; an extension to Spearman's correlation for incomplete lists; a new summary statistic called Zero Index Crossing for identifying a threshold; and finally some empirical evidence that the non-independence of the tests being performed makes multiple testing correction methods unreliable.

## Methods

We review the framework of statistical hypothesis testing for finding epistatic interactions in GWAS data (section 2.1), and briefly describe the three statistical tests compared in this paper. In section 2.2, we describe our cross-validation approach which allows us to examine the stability of SNP rankings by repeatedly splitting datasets into two halves. We also propose an extension to Spearman's correlation for incomplete lists, and suggest using the Zero Index-Crossing (ZIC) of Spearman's correlation to measure the stability of different datasets and methods.

### 2.1 Bivariate SNP analysis

This paper compares three statistical tests for association between genotype and phenotype, namely Pearson's 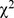 test for association, GBOOST [23], and the Gain in Sensitivity and Specificity (GSS) test [5].

Consider a population split into two disjoint subsets of *Controls* 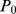 and *Cases* 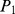 from which we have sampled relatively small subsets 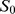 and 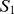 respectively. We denote each GWAS study as a collection of SNPs from a cohort of size 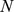 samples. We use the vector 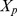 to denote the 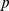-th SNP. For diploid organisms, considered in human GWAS, each SNP can take one of three genotypes depending on whether the SNP variant occurs on zero, one or both copies of an individual's relevant chromosome. We denote these genotype values as 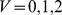 respectively, but note that 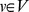 are categorical values with their value not indicating an ordinal relationship. When considering a SNP pair between the 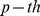 and 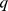-th SNP, we denote the resulting SNP pair as 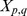, which has genotype combinations in the 9-element space 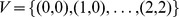.

The discrete nature of the data in SNP interaction analysis, with two possible phenotype values and three possible genotype values per SNP, allows us to summarise the occurrence of a given SNP interaction as a contingency table. In table 1, we describe such a table for an arbitrarily sized SNP interaction. Each cell indicates the occurrence of a specific genotype combination 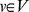 in either cases or controls. We use the notation adopted by [28] to describe the table cells where 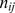 is used to denote the observed count in the cell 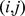. Marginal counts can be described using a standard plus convention, e.g., 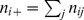 is the occurrence of all genotypes for a given phenotype, 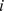. The use of contingency table based analysis is common for GWAS studies as it allows for the application of a wide variety of statistical techniques [29].

**Table 1:** 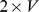
-contingency table summarising the occurrence of genotype combinations for an arbitrary SNP interaction in a case-control GWAS study.

	Genotype Frequencies				
Phenotype	1	2	…	V	Row Counts
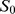	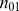	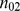	…	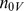	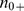
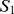	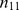	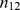	…	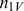	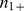
Col. Counts	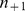	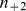	…	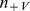	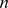

#### 2.1.1 Pearson's 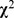 test for association

For both the univariate and bivariate case the 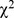 statistic can be evaluated by comparing the difference between the observed and expected frequency of Cases and Controls for each possible genotype in 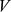:

**Definition 1** (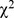 statistic):
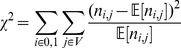

*where* 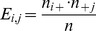


A 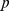-value can be calculated from the score 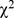 using the incomplete gamma function 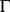 with degrees of freedom 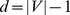 (i.e., 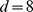 for the bivariate case) as 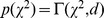.

#### 2.1.2 The GBOOST method

The GBOOST [23] method explicitly searches for interactions between SNPs by ranking candidate SNP pairs according to a likelihood ratio statistic. A logistic regression model is used to evaluate the univariate association of the 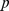-th and 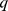-th SNPs considered in the same model, known as the *main-effects model* and described below using the notation adopted by Agresti [28] and the original BOOST paper [6].

**Definition 2** (Main-effects model):
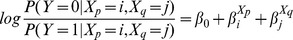



Similarly, we can construct a logistic regression model to evaluate the univariate associations of the 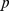-th and 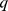-th SNPs as well as their interaction, with the resulting model known as the full model:

**Definition 3** (Full model):
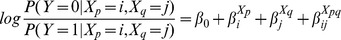



The only difference between equations defn. 2 and defn. 3 is the inclusion of an additional term in the latter to represent the interaction between the two SNPs.

The likelihood ratio test compares the association observed in the two logistic models and can determine whether modelling the interaction term on top of the univariate effects leads to a significant improvement in the fit of the resulting model. If no interaction effects exist, any association with phenotype will be captured by the main-effects association model.

Due to the computational expense of evaluating logistic regression models, the BOOST approach described by Wan et al [6] makes use of log-linear models, which are equivalent to logistic regression models, that can be derived from a contingency table of genotype frequencies combined with a two stage evaluation procedure to further improve runtime. Interested readers should consult the cited work for full details of the GBOOST method.

#### 2.1.3 Gain in Sensitivity and Specificity test

The Gain in Sensitivity and Specificity (GSS) test quantifies the ability of a pair of SNPs to segregate Cases from Controls compared to the segregation ability of the two SNPs taken individually. The classification-based approach is conceptually similar to that of Multi-Dimensional Reduction (MDR) [30], here using rigorous statistical tests to quantify the significance of improvement as opposed to the computationally-expensive cross-validation and permutation approach taken by MDR.

For each SNP or pair of SNPs, we determine a *sample prevalence mapping*, allocating to each sample the ratio of the number of Cases to the total number of Cases and Controls in the dataset which carry exactly the same genotype combination as the given sample:
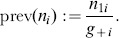
When examining a given SNP pair, we can derive three such prevalence mappings, one for the pair and two for the individual SNPs.

Each mapping can be used to construct a ROC curve: the plot of the *true positive rate* (TPR) versus the *false positive rate* (FPR). The ROC curve is easily computed from the contingency table of genotype counts for a given SNP pair. Ordering genotypes in descending order by their prevalence and taking the cumulative sum, indicates the nine TPR and FPR points corresponding to the nine thresholds of sample prevalence that have an effect on sample classification, and hence alter the ROC curve of a given SNP-pair. This method can be similarly applied to contingency tables for the SNPs individual to derive ROC curves for individual SNPs.

Let 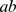 denote a pair of SNPs consisting of individual SNPs 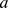 and 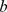. The ROC curve for the SNP pair, 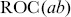 always dominates both curves for the individual SNPs 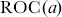 and 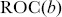 as the number of genotypes is larger, thus a finer stratification of the data is possible than that allowed by individual SNPs. For most SNP pairs, this stratification will have little effect on the ability to separate Cases and Controls but for some the difference will be significant. This improvement is the effect measured by GSS.

The area under the convex hull of 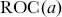 and 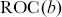 represents the null hypothesis that all Case and Control samples are drawn from the same distribution given by univariate association. A 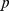-value 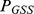 for a SNP-pair ROC curve can be derived from a Binomial distribution by computing the probability of observing higher specificity and sensitivity when drawing from the population represented by the null hypothesis.

The gain of 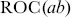 over 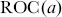 and 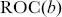 is quantified by the most significant probability (i.e., the minimum 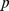-value) that a specificity and sensitivity achieved at any point in 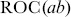 can be exceeded by random sampling of Controls and Cases from a population for which the true sensitivity and specificity are in the convex hull of 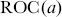 and 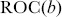. This probability is essentially dependent on the sample sizes 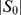 and 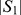 and the amount of association achieved for each single-SNP ROC curve.

A conservative measure of the gain in association can be computing by solving the following min-max optimisation of two binomials:
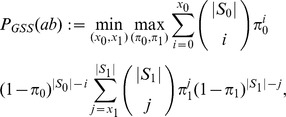
where min is over all cumulative counts 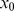 and 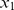 of Cases and Controls such that
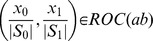
and the max is over the convex hull of the union of 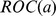 and 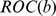:
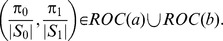
In Goudey et al. [5] we present an efficient framework known as GWIS for computing all these statistics using commodity computing resources. In all cases, bivariate contingency tables are generated as an intermediate representation and the statistics are computed from the tables. This enables us to efficiently investigate the stability of bivariate SNP analysis for the first time.

### 2.2 Cross-validation to evaluate stability

There has been recent work on gene expression data which supports the idea that features that are ranked consistently over different cross-validation folds will be more useful than features which are ranked inconsistently [13], [14]. Similar techniques have been applied to univariate GWAS analysis [15]. Here, we apply this same idea to bivariate GWAS, computing stability under the condition of two fold cross-validation, where we partition each dataset into half and compare the rankings of SNP pairs detected in each half. This is motivated by the concept of replication in biological experiments: typically, a particular discovery from one GWAS study needs to be confirmed by other GWAS studies to be accepted as a SNP showing a potential association with the given phenotype.

Given the framework of cross-validation, one still needs a measure of replication to apply over each of the folds. One straightforward option is to use the Jaccard index [31], defined as the cardinality of the intersection divided by the cardinality of the union. Note that there are many other distance metrics that may be chosen [32], however the Jaccard index is a good representative of set based distance metrics as it has been well studied. We include Jaccard index plots with our results.

The disadvantage of using the Jaccard index is it only takes into account overlap between sets and ignores the *ordering* of the lists. As we are dealing with ordered lists, and as ordering of the pairs is very important for interaction analysis, a measure incorporating the ranking of the pairs is desirable.

Measuring correlation is a natural alternative which has many desirable properties. However, it is complicated in interaction analysis as we are unable to obtain complete lists of all pairs due to space limitations. For example, the datasets used in our experiments contain approximately 500,000 SNPs, and hence around 125 billion SNP pairs. Assuming 4 bytes of information to store per SNP pair, this would result in 500 GB worth of information per dataset. As we are unable to practically store all evaluated pairs, we are forced to work with “top-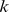
” lists, that is evaluating stability between two lists of the 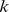 most significant pairs with 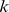 a very small fraction of the total number of possible pairs. Our measure of replication must therefore be applicable to partial top-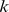 lists. Note that during computation we do not ever store the scores for all SNP pairs. Instead we store the top-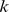 pairs in a priority queue of bounded length. In other words we compare each new pair to the worst stored score. If the new pair score is better than the worst score, the new pair is added to the top-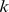 list, causing the pair with the worst score to be discarded.

For our implementation of the GSS and 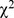 method, we can calculate the score for any missing pair and so our lists can be completed by calculating explicitly the score of any pairs missing in a list. However, the GBOOST software does not allow specification of explicit pairs to evaluate, and so we need to consider how to calculate Spearman's 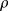 for incomplete lists.

#### 2.2.1 Spearman's 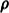 for incomplete lists

Spearman's 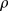 [33] is a measure of correlation between two ranked lists. Though it requires the two lists to contain the same elements, it measures the concordance between the two rankings.

**Definition 4** (Spearman's 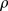) *Let* 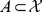 *be a ranked list with elements* 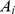 *such that* 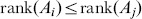 *if and only if* 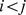 *. Let* 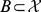 *be another such list with the same elements (but of potentially different ranks), that is* 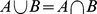 *. Spearman's* 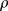 *is defined as:*

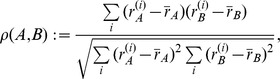

*where* 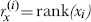 *is the rank of the item* 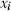 *in the list* 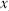 *, and* 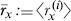 *is the average rank in the list* 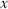.

As we do not have complete rankings over the entire domain and therefore Spearman's 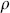 cannot be directly applied to our top-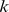 lists, we propose an extension of Spearman's 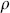 to handle partial rankings.

The key observation is that any elements in list 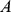 that do not appear in list 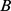 must have a rank higher than the number of elements in 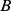. Since the elements of 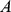 are the top-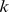 ranked elements, the elements in 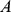 which are not in 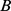 must have a rank greater than 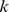. The same applies to list 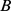. Using this observation, we can expand lists 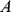 and 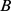 to complete rankings over the same set of elements (the union of the two lists), denoting them as 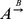 and 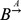 respectively. The missing values in the extension are allocated an average rank to maintain consistent fractional ranking. The extended Spearman's 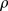 given in defn. 5 extends these lists assuming missing elements are ranked last.

**Definition 5** (Spearman's 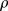 on incomplete lists) *Let* 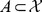 *be a ranked list with elements* 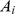 *such that* 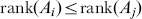 *if and only if* 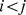 *, and* 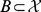 *be another such list. Define extensions* 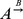 *with the elements* 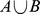 *and with ranks:*

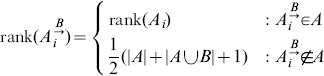

*and* 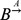 *similarly*. Spearman's 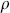 *on incomplete lists is then*

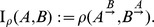



Imputing ranks of missing values as 
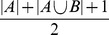
 in defn. 5 has the same average rank 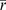 as an unambiguous list.

**Proposition 1** *Let* 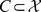 *be a list such that its elements are strictly ordered (* 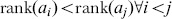 *). Consider a subset* 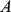 *of the ordered list* 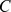 *, which retains the ordering of* 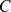 *. Let* 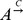 *be the extended list of* 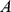 *as defined in defn. 5. Then* 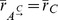.

*Proof.* As 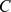 is is strictly ordered, we have
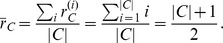
By definition of extended list 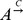:
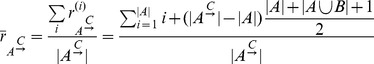


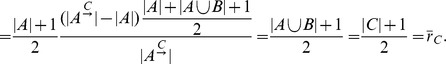



We refer to the incomplete list extension simply as Spearman's 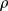 for the remainder of the paper.

#### 2.2.2 Zero Index Crossing

As we are limited to top-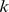 lists, the question of how to choose 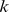 arises. Intuitively, one wishes to choose a 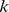 that maximises the stability of the selected pairs. Given the correlation measure presented above, a natural point to choose 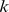 is where the correlation drops below zero (NB: negative correlations are expected under random selection of pairs as the probability of selecting the same pair twice is very small), which we denote as the zero-index crossing (ZIC). The ZIC captures the point when both lists are consistent (i.e., they contain the same elements), but the ordering is not. Given that our lists are ordered by statistical significance, this is a good choice as we wish to know which pairs are (replicably) significant, but the ordering by statistical significance has little value as it is not a substitute for effect size. ZIC can also be used as a summary statistic to compare the stability of different datasets.

**Definition 6** (Zero Index-Crossing) *Given two lists* 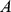 *and* 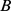 *, the Zero Index Crossing (ZIC) is given by*

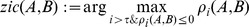

*for some threshold* 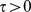.

Here we have used a threshold 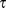 to specify a minimum size. This is necessary as the stability amongst the first few pairs is usually low, but rapidly increases after reaching a small size (see Results section). For all experiments in this paper we have chosen 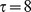.

## 3 Results & Discussion

### 3.1 Cross-Validation of Exhaustive Bivariate classification on Case–Control GWAS

Recent efficiency improvements in exhaustive bivariate GWAS analysis [23], [5] allow us to perform a comparative cross-validation study of exhaustive bivariate analysis on typical GWAS data. These results would have required weeks or months of processing using earlier methods, but in this study were mostly executed in only a few days on ordinary desktop computers using Graphics-Processing Unit (GPU) improved algorithms. This type of GWAS analysis has not previously been reported for exhaustive bivariate classification due to the excessive computing resources required.

The GSS statistic is significantly more computationally intensive than 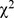 and the log-likelihood ratio tests used by GBOOST. Whereas each 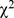 cross-validation fold took approximately 15 minutes to execute on a desktop computer, each GSS fold took approximately 6 hours. To accelerate production of results for this paper, some GSS cross-validation folds were executed on the Multi-modal Australian ScienceS Imagine and Visualisation Environment (MASSIVE) GPU cluster.

The GBOOST statistic [23] was calculated using the GPU software available for download from the author's website. Each cross-validation fold took approximately 50 minutes, meaning GBOOST lies between the two performance extremes of 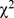 and GSS.

The Wellcome Trust Case Control Consortium (WTCCC) datasets were selected because they are publicly available and already thoroughly studied. We focused on investigating the stability of the 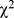, GBOOST, and GSS statistics. The WTCCC data covers seven different diseases: bipolar disease (BD), coronary artery disease (CAD), hypertension (HT), rheumatoid Arthritis (RA), type-1 diabetes (T1D), and type-2 diabetes (T2D).

Each dataset comprises of 449,471 SNPs. The number of samples vary from 4,686 (CD) to 4,901 (T1D). We computed two folds for each of ten random splits of every dataset as well as analysing the entire dataset without cross-validation, i.e., a total of 147 exhaustive bivariate analyses per statistical test. These 147 analyses were performed for 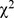, GBOOST, and GSS statistics. For each of the 147 analyses and each test, a ranked list of the most significant 1 million pairs was produced. Stability of the tests was analysed by comparison of these ranked lists. During analysis of our results, we discovered that it was necessary to prune univariately significant SNPs for the benefit of 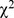. Thereafter, our entire analysis was re-run on the pruned datasets for the three statistics considered in this work. In total, 882 exhaustive bivariate analyses were completed.

This large number of analyses indicates the high performance with which exhaustive bivariate analysis of entire GWAS can now be conducted and should provided an example dispelling the myth that exhaustive bivariate analysis is a computationally infeasible procedure [7].

### 3.2 Results of : 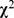 Dominated by univariate effects

**Figure 1:**
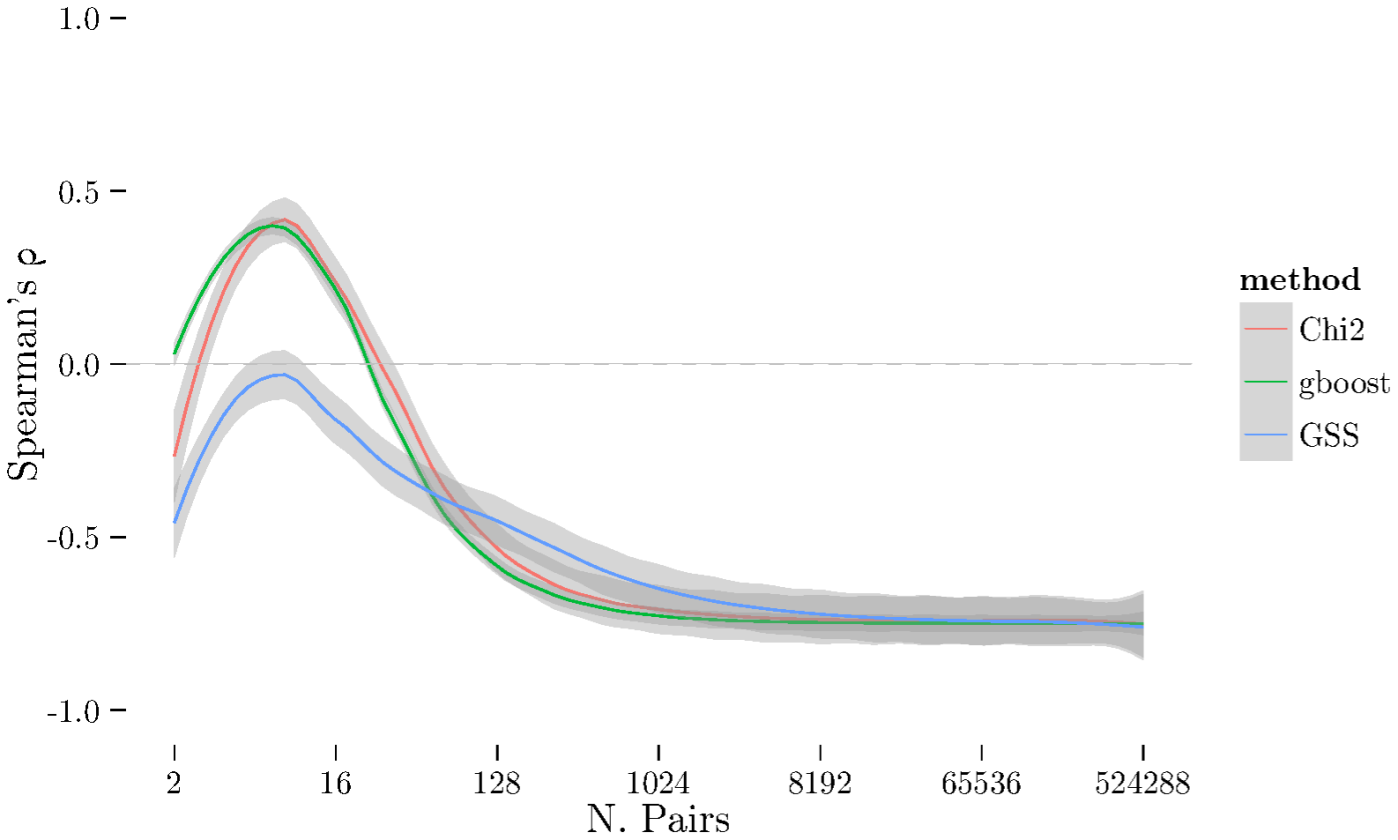
Spearman's *ρ* for all three methods (χ^2^, GSS, and GBOOST) on BD dataset. On this dataset, GSS fails to obtain a stable set of pairs on average. GBOOST and χ^2^ both have similar profiles and show similar ZIC points. Note that while the peaks for GBOOST and χ^2^ occur at approximately the same number of pairs, the higher ρ for GBOOST indicates better stability of the ordering within the stable set.

**Figure 2:**
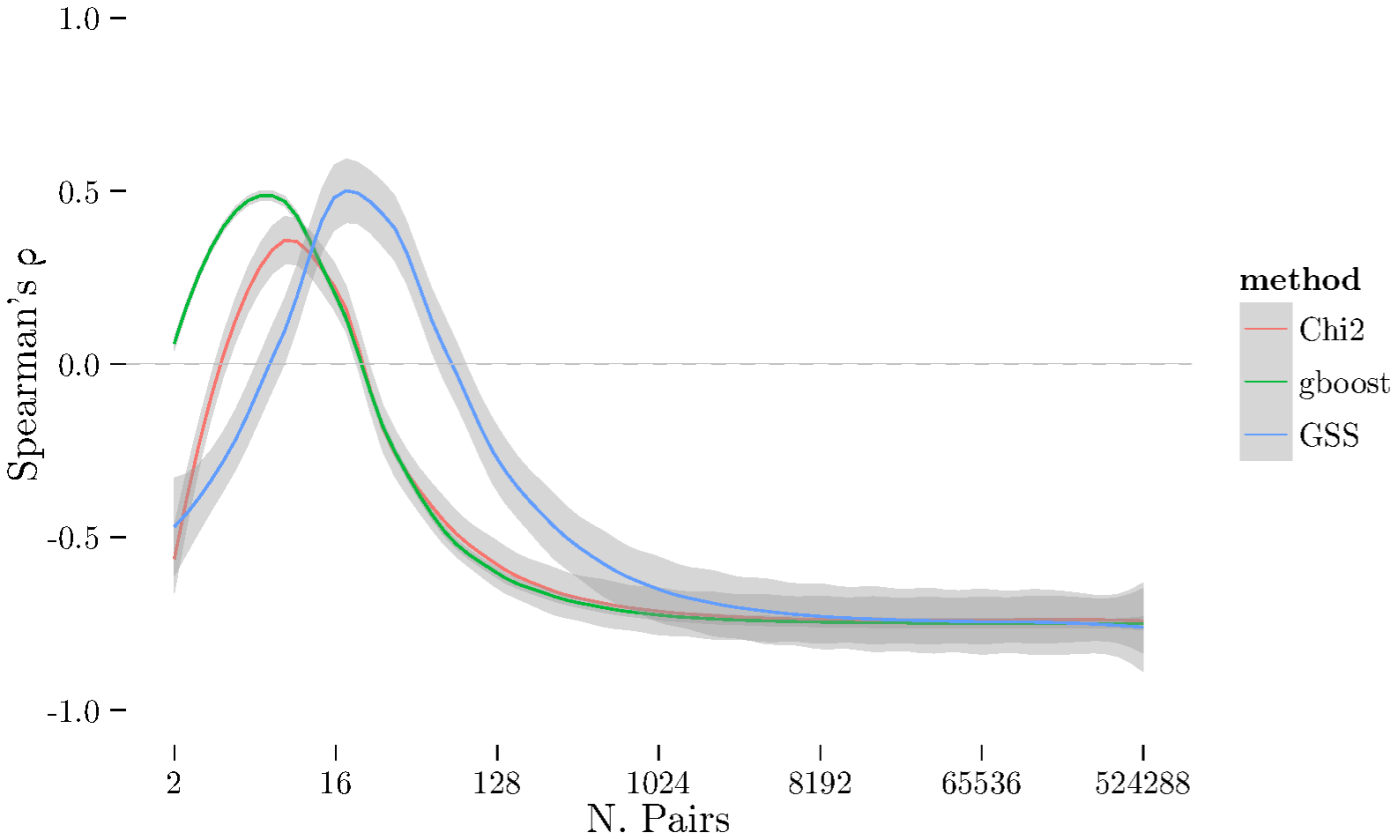
Spearman's *ρ* plot – similar to fig. 1 – for CAD dataset. Here, GSS is selecting a much larger stable set of features than χ^2^ and GBOOST, indicated by the ZIC ocurring at much larger number of pairs. Like BD, GBOOST and χ^2^ have similar profiles with GBOOST exhibiting better stability in the ordering than χ^2^.

**Figure 3:**
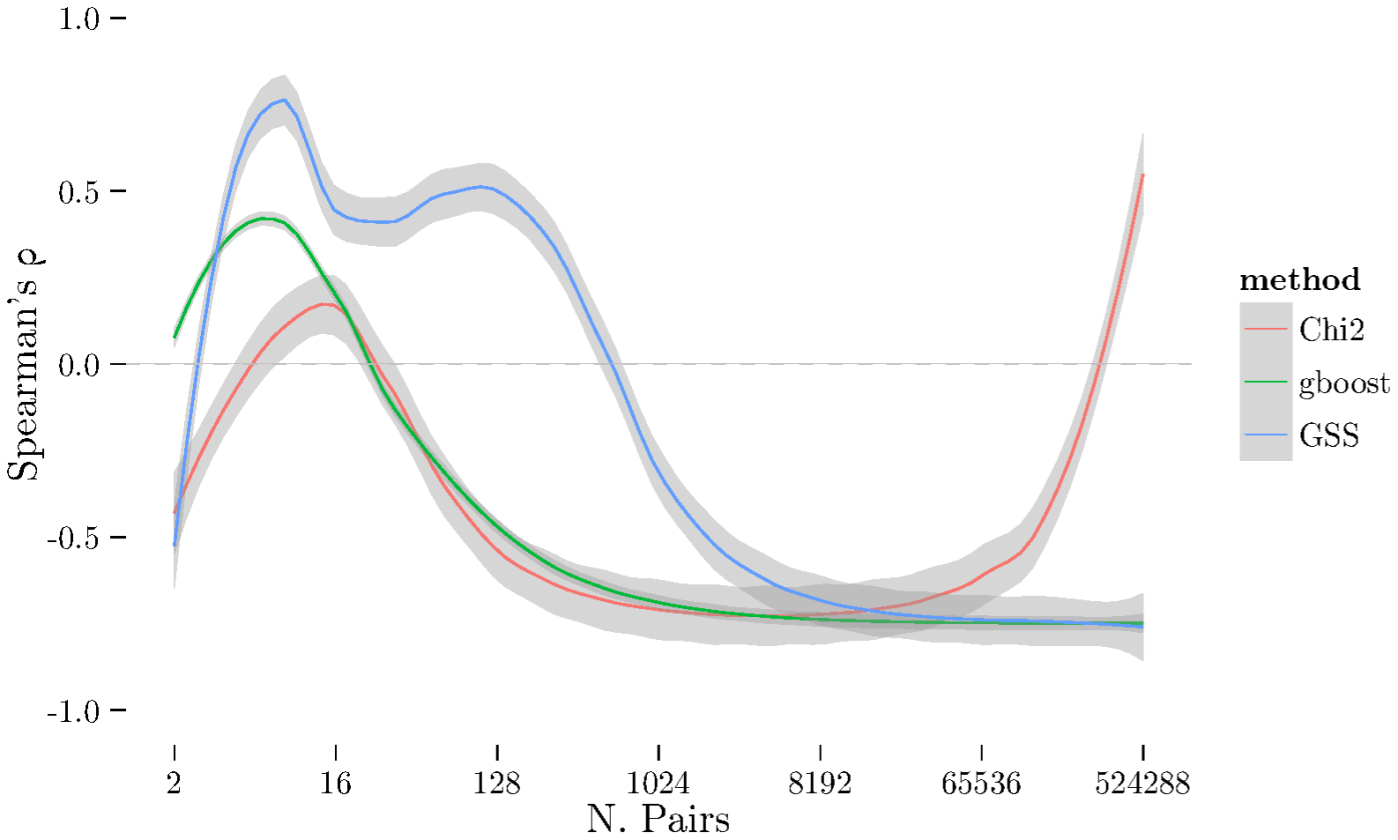
Spearman's *ρ* plot – similar to fig. 1 – for RA dataset. Here, GSS selects a significantly larger number of pairs in it's stable set while GBOOST selects relatively few. χ^2^ selects a small stable set, like GBOOST, but has curious tail behaviour where the stability increases again with a very large number of pairs. Furthermore, though GBOOST has better stability in the ordering than χ^2^, it is not significantly better than GSS unlike fig. 2.

**Figure 4:**
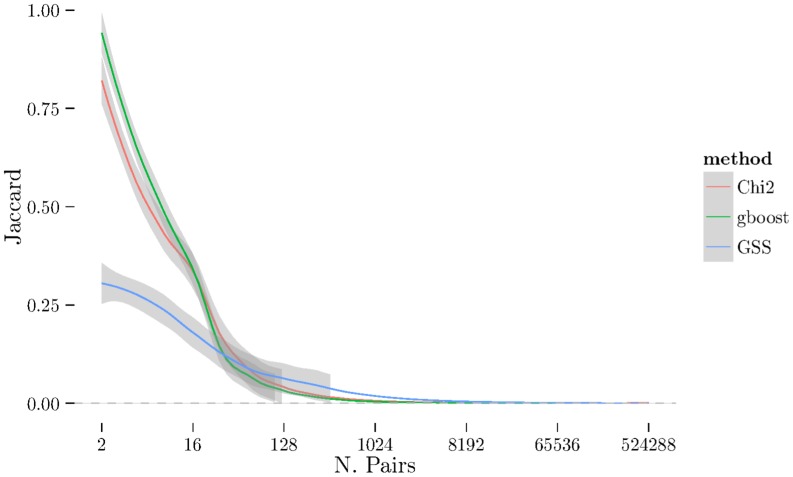
Jaccard distance for all three methods (χ^2^, GSS, and GBOOST) on BD dataset.

**Figure 5:**
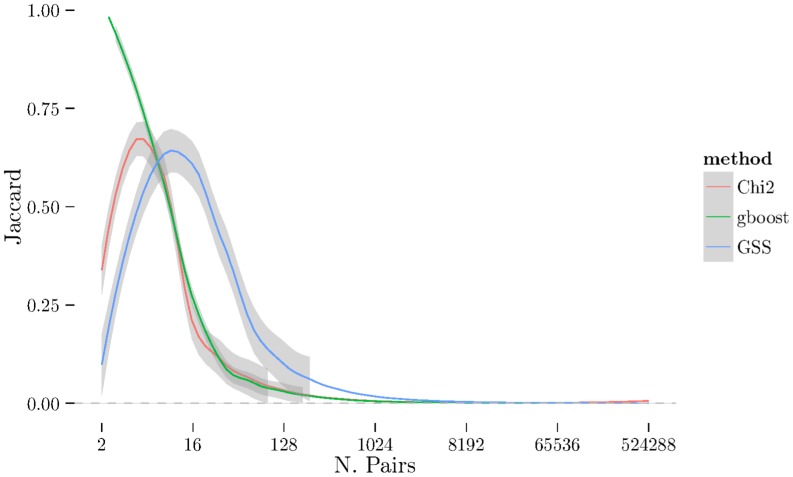
Jaccard plot for CAD dataset.

**Figure 6:**
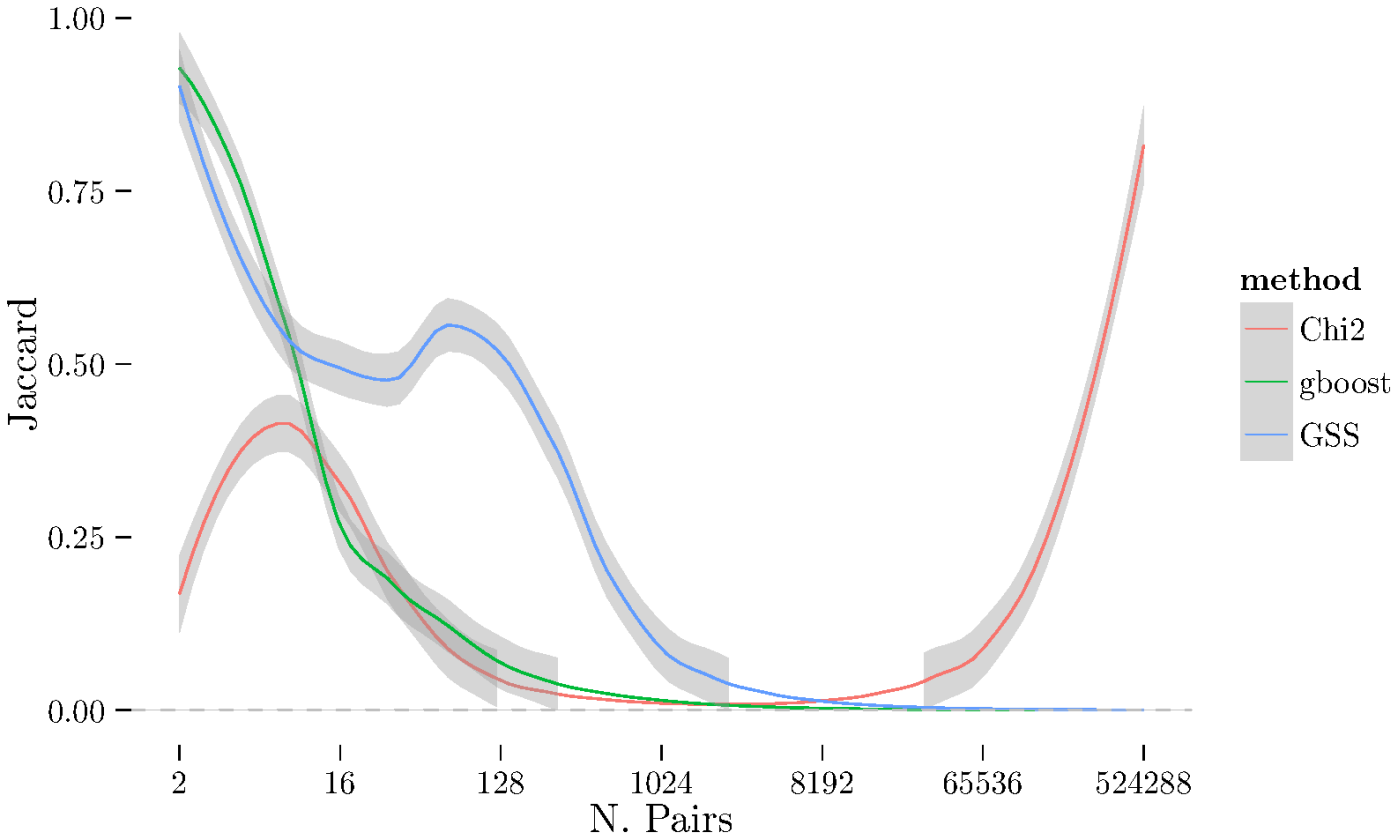
Jaccard plot for RA dataset.

We first turn to analysing the stability of 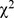 using cross-validation. The fig. 1, 2, and 3 show the results from measuring Spearman's 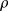 between folds using 2-fold cross-validation repeated 10 times (see Methods section) on BD, CAD, and RA. The figs. 4, 5, 6 show the same results but measured with the Jaccard index instead of Spearman's 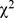. There is a very noticeable “U-shaped” artefact in three of the datasets (CD, RA, and T1D, only RA shown in fig. 3 with the others relegated to the supplementary materials) whereby the list increases to nearly 100% as the number of pairs selected approaches the total number of individual SNPs. This behaviour is also visible with the Jaccard metric (fig. 6).

**Figure 7:**
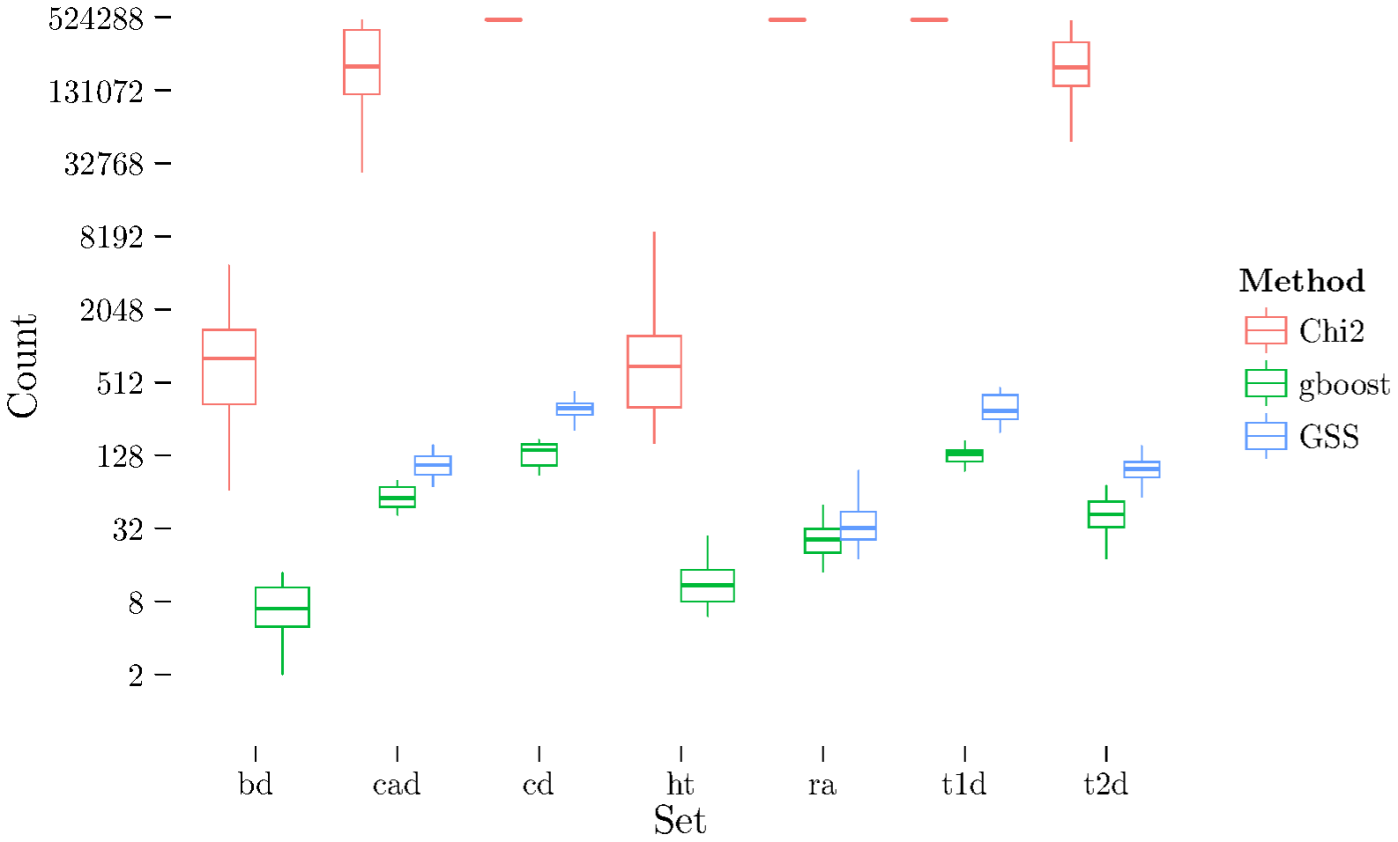
Boxplot of the number of pairs involving a univariately significant SNP (by univariate χ^2^ test) for each dataset and method. The extreme high counts for CD, RA, and T1D datasets for the χ^2^ test indicate that these datasets are strongly confounded by extremely large hubs driven by main effects. These datasets also demonstrate the U-shaped tail behaviour of χ^2^ (e.g., fig. 3), indicating the high stability is only caused by these very large stable hubs. GSS is not shown on the BD or HT datasets as there were no pairs associated with a univariately signifiant SNP.

One hypothesis of the cause of this is that the lists of detected SNP pairs are being dominated by SNPs with a strong univariate disease association: when ranked by 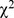, SNPs with a strong disease association often pair with *every* other SNP to form a strong bivariate pair. In this case, most of the association of the SNP pair is a function of the association of one SNP. We call the single strong SNP a “hub”, as the degree of this vertex in an interaction graph would be very high. A hub SNP can also be defined as a SNP with very high frequency in the ranked pairs lists. To investigate this hypothesis, we calculated the number of pairs in a given list that involve a univariately significant SNP. The results are shown in fig. 7.

Two observations are evident from this figure, the first is that that the hubs present in the lists are dominated by univariately significant SNPs, and the second is that simple bivariate 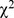 association analysis is in general confounded by main effects. Note that this observation does not necessarily apply to derivatives of Pearson's 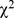 test for association. The first claim is evidenced by the total connectedness of all pairs with a univariately significant SNP for the CD, RA, and T1D datasets 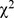 in fig. 7. The second claim is supported by the observation that on all datasets has significantly more pairs associated with univariate SNPs than both GBOOST and GSS.

**Figure 8:**
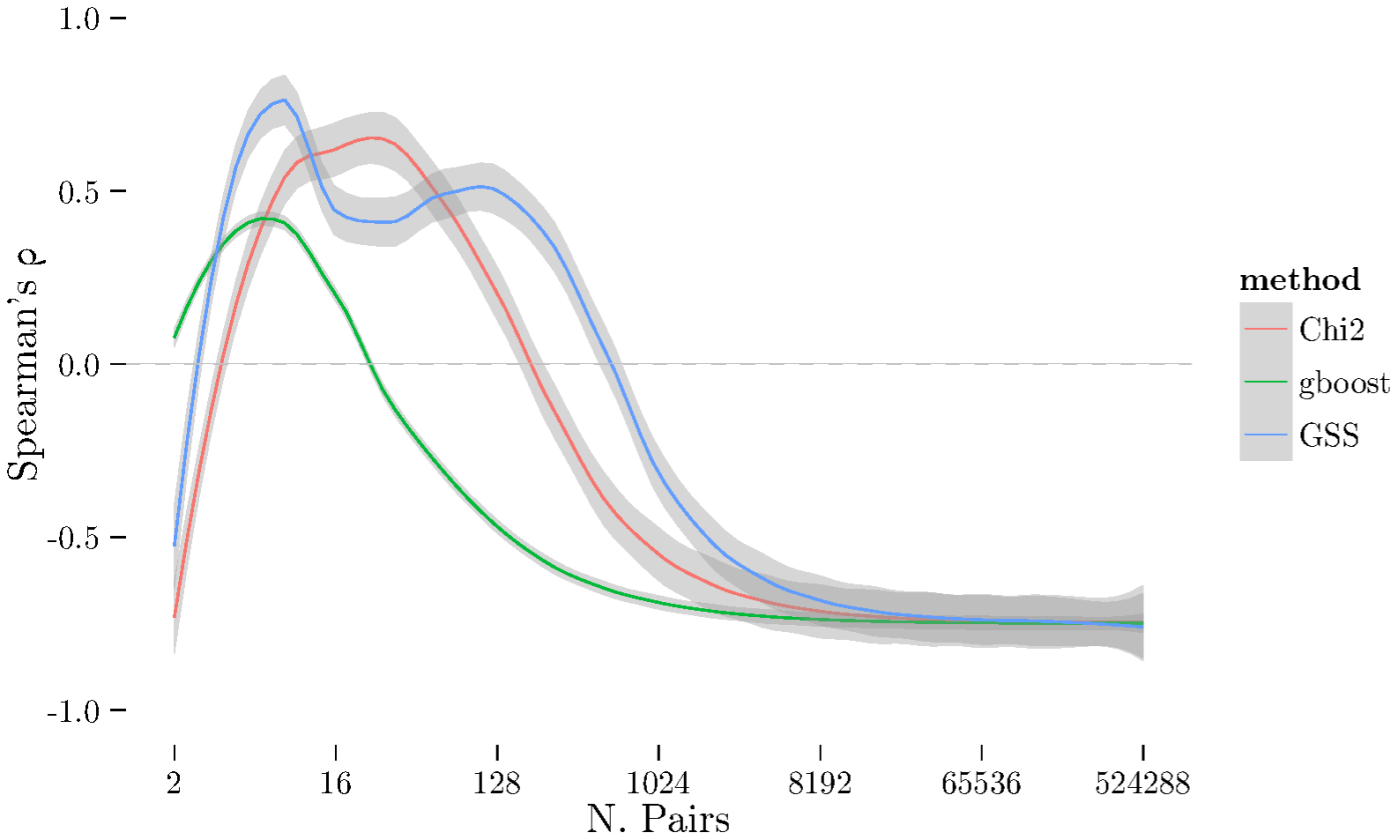
Spearman's *ρ* plot for pruned RA dataset – similar to fig. 1. After dataset pruning (by removing SNPs significant under a univariate χ^2^ test) we see the curious tail behaviour of χ^2^ is gone. The GSS profile remains similar to fig. 3. This suggests the tail effect is caused by main effects confounding the χ^2^ interaction test.

**Figure 9:**
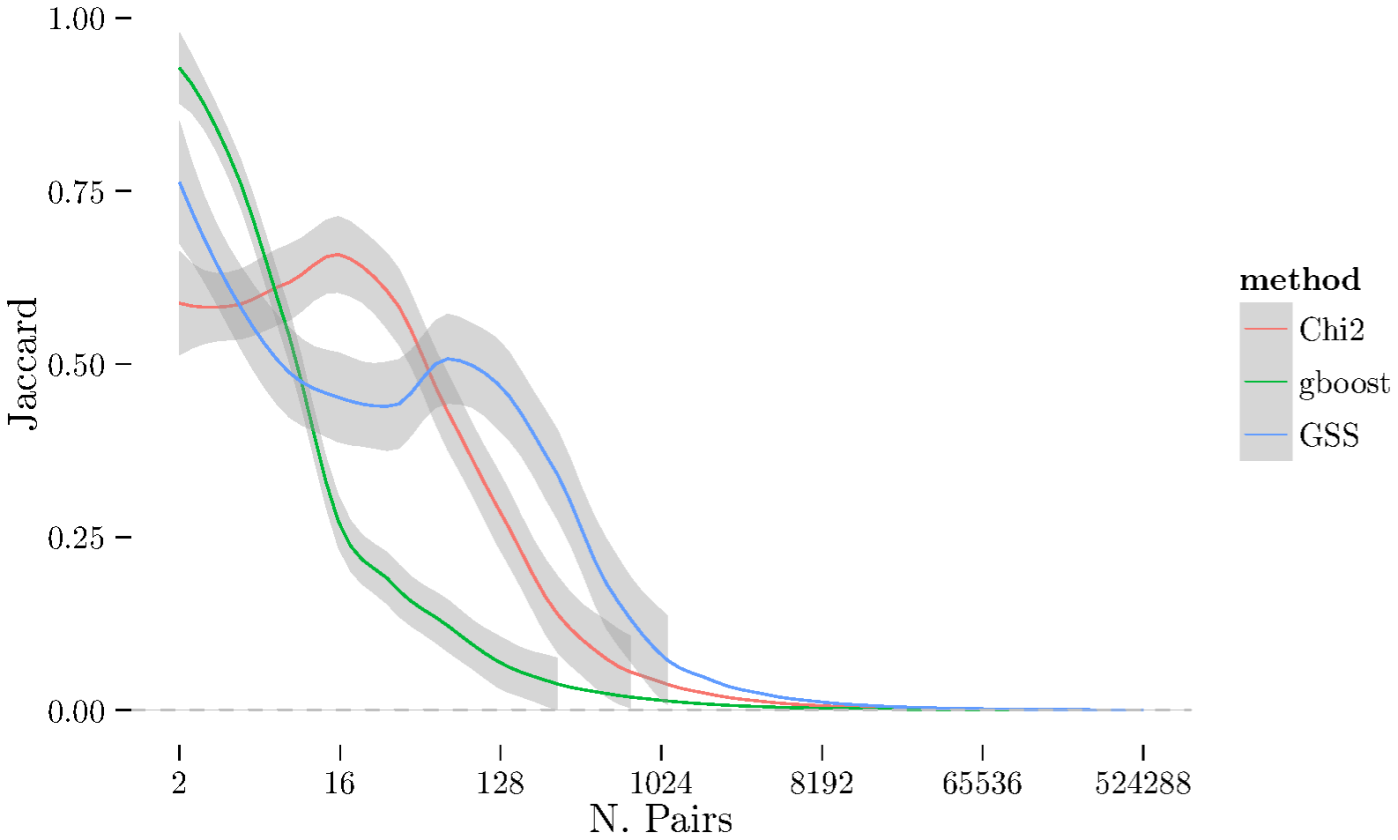
Jaccard plot for pruned RA dataset.

To address the first problem, we pruned the WTCCC datasets using PLINK [34], discarding all univariately significant SNPs according to a univariate 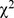 test at the Bonferroni level (
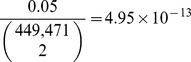
). This resulted in removing 7 SNPs from BD, 37 from CAD, 71 from CD, 6 from HT, 17 from RA, 87 from T1D, and 27 from T2D. After pruning, we recomputed the profiles for GSS and 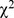 on the RA datasets as shown in figs. 8 and 9. Here we see that while GSS has a very similar profile to before pruning (figs. 3 and 6), 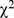 has changed dramatically and now produces much longer and more stable list. Furthermore, the U-shaped tail behaviour observed in figs. 3 and 6 is no longer present.

Although pruning is able to address the domination of the 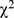 lists by strong univariate SNPs, there is no easy corrective method that can be applied to reduce the subtle main effect bias observed earlier. In addition, it is quite possible that SNPs with univariate association also play a role in bivariate or higher-order interactions. In fact, it may be more likely that these SNPs participate in interactions, although this is currently unknown. Removing these SNPs from the dataset prevents these pairs from being properly evaluated in higher order analysis. To address these issues completely, alternative statistics are needed that explicitly take main effects into account. GSS and GBOOST are two such statistics, results of which are discussed below.

### 3.3 Stability of GSS and GBOOST

We now turn our attention to the GSS statistic. It is immediately noticeable that the U-shaped behaviour evident with 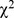 is non-existent. This is expected as earlier we demonstrated the U-shape was due to domination by univariately strong SNPs. As the GSS explicitly models the gain over main effects such confounding is not possible. Indeed, fig. 7 verifies this claim as GSS has significantly smaller hubs connected to univariately significant SNPs.

GBOOST also does not exhibit the U-shaped behaviour. Furthermore, fig. 7 shows the size of the univariate hubs are somewhat similar to GSS and are not large as with. Like GSS, GBOOST also explicitly models the improvement over main effects and so is not affected by the main effect bias. These results show that both GSS and GBOOST successfully discount for strong univariate effects and target bivariate effects.

### 3.4 Stability differences between 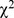, GSS, and GBOOST

To quantify more precisely the comparative stability of both the three statistics, we calculated the ZIC (see section 2.2.2) for each dataset on both pruned and unpruned data, with results shown in table 2. Recall that ZIC is a summary of the stability of detected SNP pairs with a larger ZIC indicating increased stability. Our first observation is that GSS has significantly better stability than 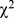 on the unpruned RA, T1D and CD datasets, and is no worse than 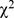 on any unpruned dataset. Comparing GSS to GBOOST, on the unpruned datasets we find that GSS has significantly better stability than GBOOST on the same three datasets. This consistent selection of significantly more pairs suggests that GSS is capturing some set of pairs that cannot be detected using the GBOOST statistics. However, as discussed below, it also seems the converse is also true and GBOOST detects pairs that are not detected by GSS.

**Table 2:** Stability as measured by the Zero Index Crossing (ZIC) (see defn.6).

	ZIC								
	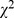			GSS			GBoost		
Dataset	Lower 95% CI	Mean	Upper 95% CI	Lower 95% CI	Mean	Upper 95% CI	Lower 95% CI	Mean	Upper 95% CI
bd	22.3	27.7	33.1	5.3	11.2	17.1	20.3	23.7	27.1
cad	16.2	21.5	26.8	7.8	39.2	70.6	20.6	20.9	21.2
cd	6.7	11.2	15.7	69.4	74.4	79.4	11.0	11.0	11.0
ht	50.1	54.4	58.7	−10.8	42.6	96.0	29.2	30.2	31.2
ra	12.4	26.1	39.8	489.4	519.4	549.4	18.9	24.4	29.9
t1d	13.6	18.1	22.6	−1.6	49.7	101.0	24.1	26.6	29.1
t2d	8.0	8.0	8.0	6.5	71.1	135.7	41.5	45.2	48.9
bdp	22.3	27.7	33.1	5.3	11.2	17.1	20.3	23.7	27.1
cadp	20.3	23.5	26.7	7.8	39.2	70.6	20.6	20.9	21.2
cdp	24.5	35.4	46.3	65.7	70.8	75.9	11.0	11.0	11.0
htp	50.4	54.5	58.6	−10.8	42.6	96.0	29.2	30.2	31.2
rap	81.6	161.0	240.4	488.6	517.5	546.4	18.9	24.4	29.9
t1dp	25.6	35.8	46.0	−1.6	49.7	101.0	24.3	26.8	29.3
t2dp	35.2	63.5	91.8	6.5	71.1	135.7	41.5	45.2	48.9

GSS has significantly better stability on the unpruned RA, T1D and CD datasets, and is no worse than 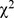 on any unpruned dataset. GSS has significantly better stability than GBOOST on the same three datasets. Pruning has almost no effect on the ZIC obtained with GBOOST, and no significant effect on GSS.

Second, the ZIC demonstrates clearly that pruning does not degrade the stability 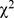 of among the top ranked pairs for any datasets, and in the case of RA there is a significant increase in stability. After pruning, the stability of 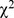 becomes comparable to GSS, albeit with the limitations already discussed. GSS is still significantly more stable 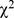 than for both the RA and CD datasets, but is significantly less stable than 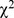 for BD.

Third, we observe that both GBOOST and 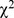 have very similar stable set sizes for all datasets. This is somewhat surprising as the two methods are fundamentally different, as GBOOST specifically looks for deviations from the additive model, but does 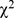 not discount univariate effects at all.

**Figure 10:**
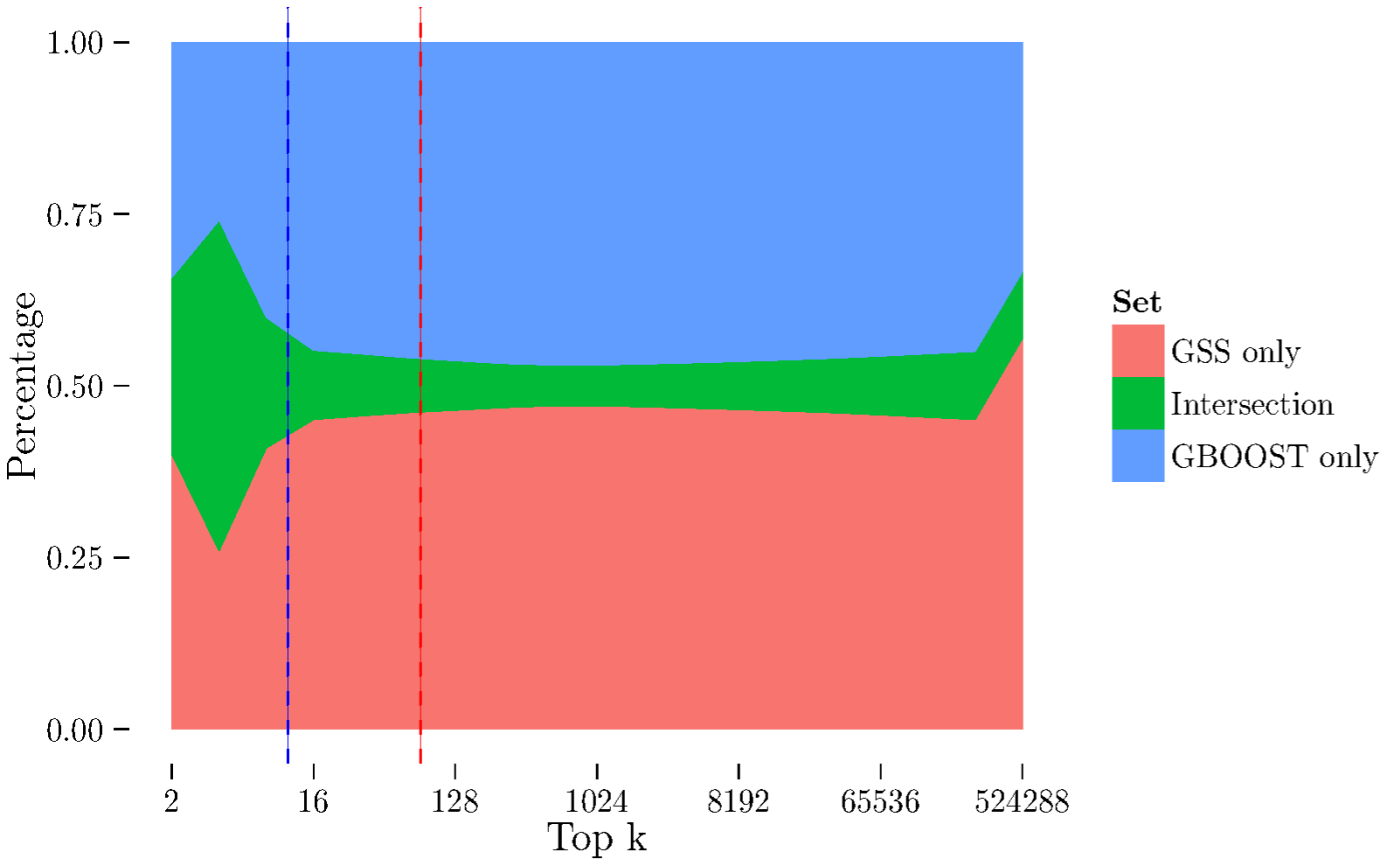
The overlap between SNP pairs found by GSS and GBOOST is plotted for various values of k. The vertical axis is scaled by the size of the union of both sets. The blue, green and red sections show respectively: the percentage of pairs which are found by GSS only, common to both methods, and found by GBOOST only. The vertical dashed red and blue lines are the ZIC values for GSS and GBOOST respectively. In all 7 datasets the relative size of the intersection set for both methods peaks at a k lower than *max*(*k^ZIC^* ^–*GBOOST*^, *k^ZIC^* ^–*GSS*^). Since both methods are intended to capture a similar type of interaction and do not have a substantial intersection at higher k, this supports the idea that ZIC is a useful heuristic. Over all values of k for all datasets, the max intersection set size ranges from 0.2 to 0.4. Despite some agreement, the fact that both methods are able to reliably select independent sets of pairs suggests that there are fundamental differences between the pairs selected by both methods. These intersection plots are shown for all datasets in the supplement. The result for the CD dataset is shown here as an example.

Given that GBOOST and GSS attempt to quantify the level of improvement in association of a SNP pair compared to its individual SNPs, the obvious question is whether they pick the same pairs. To gain some insight into this question, we plotted the relative size of the intersection set between top pairs picked by GSS and GBOOST for various values of 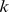 (see fig. 10 for an example). In all datasets the peak intersection set size was between 20 and 40% of 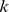. Given the very large number of candidate pairs, this suggests there are types of interaction that are reliably detected by both methods. However, for all but one dataset, the peak intersection size occurs at a lower 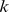 than the ZIC values of the two methods. This suggests that both methods are able to reliably select pairs of SNPs that are not reliably highly ranked by the other method, indicating GSS and GBOOST both targeting different types of interactions. Further analysis and description of the classes of interactions mutually and uniquely detected by each method is beyond the scope of this paper.

### 3.5 Multiple Testing Correction

A common way to select significant SNP pairs is to perform multiple testing correction and to select those pairs above a 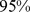 level of significance. Correction for family-wise error rate (FWER) is obtained using the Bonferroni correction, which is considered quite conservative. When the hypothesis tests are independent, Bonferroni correction is tight, since any one of the multiple tests may be rejected with equal probability [35]. However, large correlations between SNPs are known to exist in GWAS, and hence the conducted tests will not be independent causing the Bonferroni correction to be overly stringent. As an alternative, correction for false discovery rate (FDR) using the Benjamini–Hochberg procedure has been widely used for high throughput data as it is less stringent at the cost of allowing a small proportion of false discoveries.

**Figure 11:**
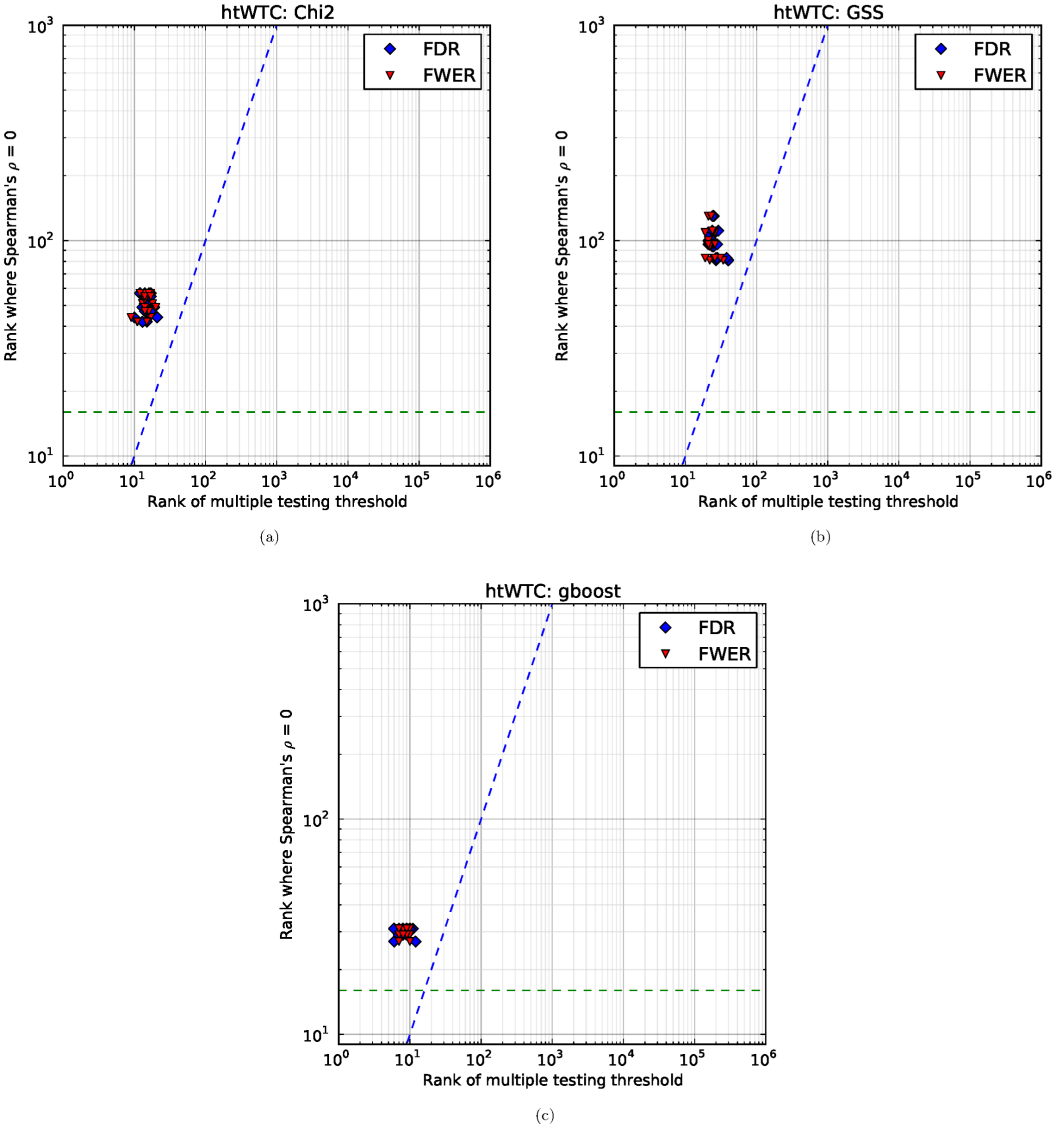
Comparing multiple testing correction and stability. On the horizontal axis, we have the rank at which the pair falls below the multiple testing correction threshold. On the vertical axis, we have the rank at which ZIC occurs. The dashed blue line is the diagonal, representing equal ranks for both ZIC and FWER/FDR. The green dashed line represents the floor for ZIC (we do not search for ZIC lower than this point due to noise). The scatter plot shows points which are above the diagonal, which means that the number of SNP pairs which are stable is consistently higher than both FWER and FDR correction.

**Figure 12:**
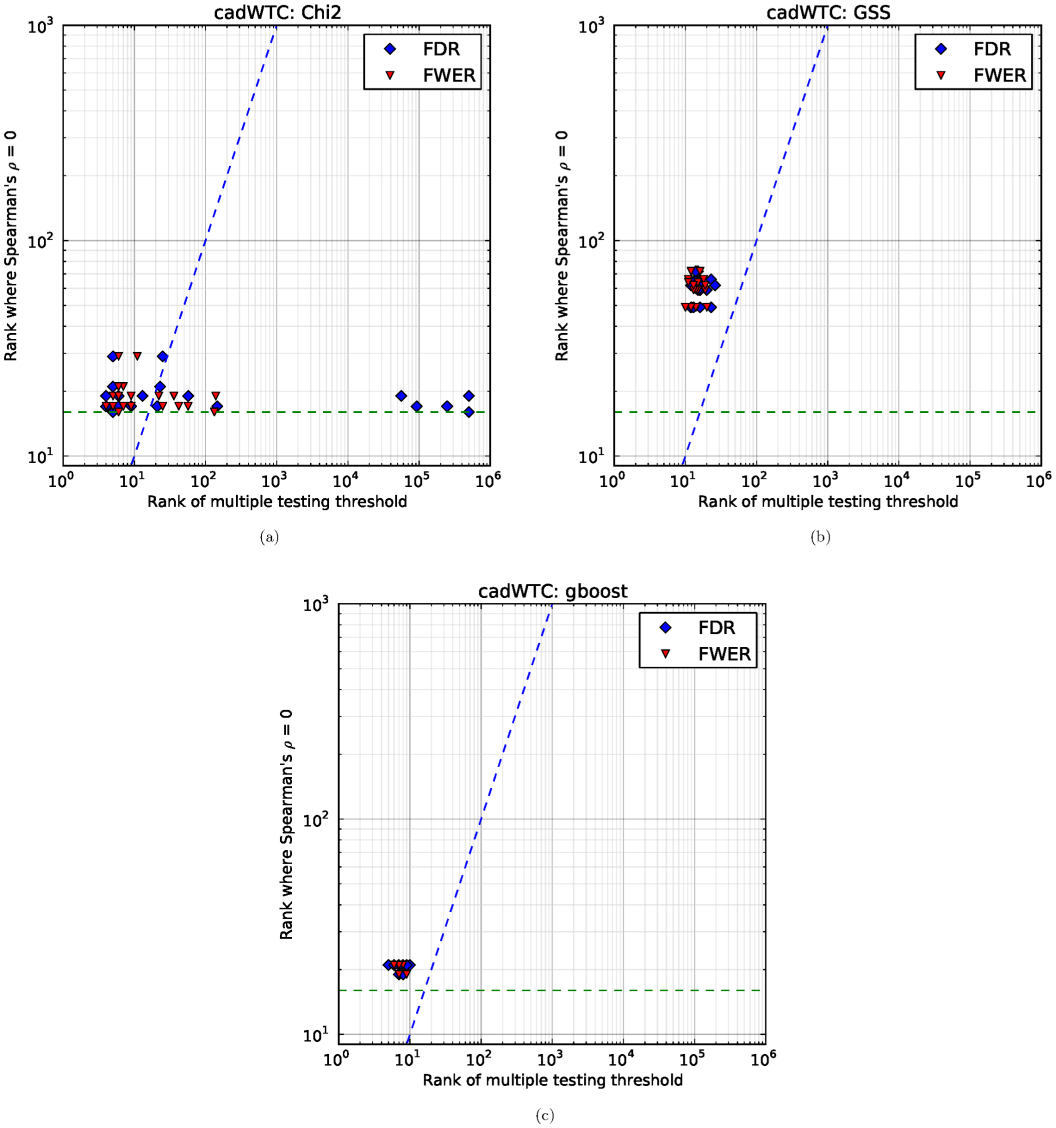
Comparing multiple testing correction and stability. Plot axes are the same as in fig. 11. The χ^2^ hypothesis test exhibits wildly differing values for FDR in different splits of the dataset, which means that the number of significant SNP pairs cannot be stably determined for this dataset. Note that we only retain the top 500,000 SNP pairs in our calculations hence the points on the right actually mean that more than 500,000 pairs pass multiple testing correction (which is highly implausible for these datasets). Observe that ZIC has only a small variance between different splits of the data. Furthermore, observe that the GSS statistic does not exhibit the large variance in multiple testing correction values. a: χ^2^; b: GSS; c: GBOOST.

However, in our setting of bivariate SNPs, the hypothesis tests are highly dependent on each other. In fact, each test is dependent on all other tests as we consider all pairs in an exhaustive fashion. In this section, we compare the number of SNP pairs that pass multiple testing correction with the ZIC, the number of SNP pairs that have positive value of our extension of Spearman's 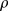. Hence we empirically check how many stable SNP pairs are found in the seven WTCCC datasets, as well as computing the Bonferroni correction and Benjamini–Hochberg procedure. For each of the 20 subsets of pairs coming from our two-fold cross-validation conducted 10 times, we compute the number of SNP pairs that pass multiple testing correction, i.e., the rank of the SNP pair which is just at the threshold. For each of the 10 splits, we compute ZIC, and plot it against the multiple testing correction values. The results for hypertension and coronary artery disease are shown in fig. 11 and 12 respectively. The results for the other five diseases are available in the supplement.

As can be seen from fig. 11, our proposed index (ZIC) exhibits good behaviour (i.e., low variance) and is comparable to using Bonferroni correction or the Benjamini–Hochberg procedure on this dataset. This provides evidence that stability is a good criteria for selecting features. As observed in section 3.4, GSS has higher ZIC values than 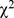, and fig. 11 shows that this effect is also corroborated by the multiple testing correction methods. As expected, the Benjamini–Hochberg procedure is less conservative and selects more SNP pairs compared to Bonferroni correction.

Furthermore, ZIC is consistently above the diagonal all three statistical tests, which means that the multiple testing correction approaches are conservative in comparison to the number of replicable features. This behaviour is to be expected as the multiple testing correction approaches are close to optimal when the hypothesis tests are independent, but the bivariate tests that we consider are not independent.

The results in fig. 12b show similar consistency and overall improvement for the GSS statistic compared to the multiple testing correction methods. However, the results in fig. 12a for the 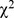 statistic show that FDR wildly varies. This could be due to a small number of individuals with a particular genotype that are highly correlated with phenotype. Hence if the split contains these individuals, it results in many selected pairs.

## Conclusions

We investigated the stability of SNP pairs found using bivariate hypothesis testing. Stability was investigated by repeatedly splitting the GWAS datasets in half, evaluating and ranking all pairs in each half and then estimating the correlation between rankings observed in both halves. These analyses were conducted using GBOOST and the GWIS platform for 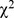 and GSS statistics. All processing was executed on commodity desktop computer hardware with general-purpose graphics processing units (GPGPU expansion cards).

For the 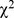 and GSS statistics we were able to compute true ranks for all SNP pairs in each split and fold of the data. However, for GBOOST it was necessary to impute ranks for pairs not assigned a significant score in a particular dataset split or fold. We proposed an extension to Spearman's 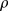 that computes the correlation between two partial top-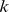 lists of ranked items without a common union. This leads to a natural measure of stability when comparing incomplete ranked outputs of large datasets. Furthermore, we proposed the Zero Index Crossing (ZIC) as a way to choose 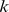 for which the selected putative SNP pairs are considered to be stable. We suggest that ZIC can also be used as a summary statistic to compare the stability of different datasets and methods.

Using Spearman's 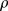 and ZIC, we evaluated the stability of 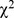, GSS, and GBOOST statistics for ranking bivariate SNP pairs. We empirically investigated stability using 10 repeats of 2 fold cross-validation on seven Case–Control GWAS datasets from the WTCCC. This is the first report of a cross-validation study on exhaustive bivariate interaction.

We found the 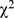 test for association rankings were highly confounded by strong univariate SNPs, resulting in a surprising “U”-shaped curve for Spearman's 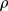. This “U”-shaped effect was reduced when univariately significant SNPs were removed from the dataset, confirming the source of the confounding factor.

The regression based GBOOST, and the recently proposed statistical test GSS, were unaffected by univariate bias. Both these methods explicitly select via the level of improvement in association for pairs of SNPs, over individual SNPs.

The GSS test was successfully able to rank a larger set of SNP-pairs with higher or equal stability than both 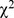 and GBOOST in both pruned and original datasets, with the exception of the BD dataset. Comparison of the SNP-pairs detected by GSS and GBOOST shows that both methods reliably detect a small set of mutual pairs, i.e., the intersection between the stable sets for GSS and GBOOST contained a small set of pairs.

By comparing ZIC with the thresholds chosen by multiple testing correction, we observe, for the GSS and GBOOST statistics, that ZIC behaves similarly to Bonferroni correction and the Benjamini–Hochberg procedure. Interestingly, ZIC is consistent for different splits of the data for the 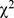 statistic but the Benjamini–Hochberg procedure seems to have large variance, suggesting that it may be inappropriate for this data. GSS achieved the largest average ZIC in our benchmark.

We conclude that the 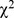 test was not able to detect bivariate effects without additional compensation for univariate effects. In contrast, tests such as GSS and GBOOST that explicitly model the improvement over individual SNPs are better able to stably select candidate pairs for further analysis.

## Supporting Information

Figure S1 **As** **fig. 1** **for Chron's Disease (CD).**
(EPS)

Figure S2 **As** **fig. 1** **for Hypertension (HT).**
(EPS)

Figure S3 **As** **fig. 1** **for Type-1 Diabetes (T1D).**
(EPS)

Figure S4 **As** **fig. 1** **for Type-2 Diabetes (T2D).**
(EPS)

Figure S5 **As** **fig. 8** **for BD.**
(EPS)

Figure S6 **As** **fig. 8** **for CAD.**
(EPS)

Figure S7 **As** **fig. 8** **for CD.**
(EPS)

Figure S8 **As** **fig. 8** **for HT.**
(EPS)

Figure S9 **As** **fig. 8** **for T1D.**
(EPS)

Figure S10 **As** **fig. 8** **for T2D.**
(EPS)

Figure S11 **As** **fig. 8** **for T2D.**
(EPS)

Figure S12 **As** **fig. 4** **for CD.**
(EPS)

Figure S13 **As** **fig. 4** **for HT.**
(EPS)

Figure S14 **As** **fig. 4** **for T1D.**
(EPS)

Figure S15 **As** **fig. 4** **for T2D.**
(EPS)

Figure S16 **As** **fig. 9** **for BD.**
(EPS)

Figure S17 **As** **fig. 9** **for CAD.**
(EPS)

Figure S18 **As** **fig. 9** **for CD.**
(EPS)

Figure S19 **As** **fig. 9** **for HT.**
(EPS)

Figure S20 **As** **fig. 9** **for T1D.**
(EPS)

Figure S21 **As** **fig. 9** **for T2D.**
(EPS)

Figure S22 **As** **fig. 10** **for BD.**
(EPS)

Figure S23 **As** **fig. 10** **for CAD.**
(EPS)

Figure S24 **As** **fig. 10** **for HT.**
(EPS)

Figure S25 **As** **fig. 10** **for RA.**
(EPS)

Figure S26 **As** **fig. 10** **for T1D.**
(EPS)

Figure S27 **as** **fig. 10** **for T2D.**
(EPS)

Figure S28 **As** **fig. 11** **for BD – χ^2^.**
(EPS)

Figure S29 **As** **fig. 11** **for BD – GSS.**
(EPS)

Figure S30 **As** **fig. 11** **for BD – GBOOST.**
(EPS)

Figure S31 **As** **fig. 11** **for BD pruned – χ^2^.**
(EPS)

Figure S32 **As** **fig. 11** **for BD pruned – GSS.**
(EPS)

Figure S33 **As** **fig. 11** **for BD pruned – GBOOST.**
(EPS)

Figure S34 **As** **fig. 11** **for CAD pruned – χ^2^.**
(EPS)

Figure S35 **As** **fig. 11** **for CAD pruned – GSS.**
(EPS)

Figure S36 **As** **fig. 11** **for CAD pruned – GBOOST.**
(EPS)

Figure S37 **As** **fig. 11** **for CD – χ^2^.**
(EPS)

Figure S38 **As** **fig. 11** **for CD – GSS.**
(EPS)

Figure S39 **As** **fig. 11** **for CD – GBOOST.**
(EPS)

Figure S40 **As** **fig. 11** **for CD pruned – χ^2^.**
(EPS)

Figure S41 **As** **fig. 11** **for CD pruned – GSS.**
(EPS)

Figure S42 **As** **fig. 11** **for CD pruned – GBOOST.**
(EPS)

Figure S43 **As** **fig. 11** **for HT pruned – χ^2^.**
(EPS)

Figure S44 **As** **fig. 11** **for HT pruned – GSS.**
(EPS)

Figure S45 **As** **fig. 11** **for HT pruned – GBOOST.**
(EPS)

Figure S46 **As** **fig. 11** **for RA – χ^2^.**
(EPS)

Figure S47 **As** **fig. 11** **for RA – GSS.**
(EPS)

Figure S48 **As** **fig. 11** **for RA – GBOOST.**
(EPS)

Figure S49 **As** **fig. 11** **for RA pruned – χ^2^.**
(EPS)

Figure S50 **As** **fig. 11** **for RA pruned – GSS.**
(EPS)

Figure S51 **As** **fig. 11** **for RA pruned – GBOOST.**
(EPS)

Figure S52 **As** **fig. 11** **for T1D – χ^2^.**
(EPS)

Figure S53 **As** **fig. 11** **for T1D – GSS.**
(EPS)

Figure S54 **As** **fig. 11** **for T1D – GBOOST.**
(EPS)

Figure S55 **As** **fig. 11** **for T1D pruned – χ^2^.**
(EPS)

Figure S56 **As** **fig. 11** **for T1D pruned – GSS.**
(EPS)

Figure S57 **As** **fig. 11** **for T1D pruned – GBOOST.**
(EPS)

Figure S58 **As** **fig. 11** **for T2D – χ^2^.**
(EPS)

Figure S59 **As** **fig. 11** **for T2D – GSS.**
(EPS)

Figure S60 **As** **fig. 11** **for T2D – GBOOST.**
(EPS)

Figure S61 **As** **fig. 11** **for T2D pruned – χ^2^.**
(EPS)

Figure S62 **As** **fig. 11** **for T2D pruned – GSS.**
(EPS)

Figure S63 **As** **fig. 11** **for T2D pruned – GBOOST.**
(EPS)
